# Exome Sequencing Analysis Identifies Rare Variants in *ATM* and *RPL8* That Are Associated With Shorter Telomere Length

**DOI:** 10.3389/fgene.2020.00337

**Published:** 2020-04-30

**Authors:** Ashley van der Spek, Sophie C. Warner, Linda Broer, Christopher P. Nelson, Dina Vojinovic, Shahzad Ahmad, Pascal P. Arp, Rutger W. W. Brouwer, Matthew Denniff, Mirjam C. G. N. van den Hout, Jeroen G. J. van Rooij, Robert Kraaij, Wilfred F. J. van IJcken, Nilesh J. Samani, M. Arfan Ikram, André G. Uitterlinden, Veryan Codd, Najaf Amin, Cornelia M. van Duijn

**Affiliations:** ^1^Department of Epidemiology, Erasmus MC University Medical Center Rotterdam, Rotterdam, Netherlands; ^2^SkylineDx B.V., Rotterdam, Netherlands; ^3^Department of Cardiovascular Sciences, University of Leicester, Leicester, United Kingdom; ^4^Department of Internal Medicine, Erasmus MC University Medical Center Rotterdam, Rotterdam, Netherlands; ^5^NIHR Leicester Biomedical Research Centre, Glenfield Hospital, Leicester, United Kingdom; ^6^Center for Biomics, Erasmus MC University Medical Center Rotterdam, Rotterdam, Netherlands; ^7^Department of Neurology, Erasmus MC University Medical Center Rotterdam, Rotterdam, Netherlands; ^8^Nuffield Department of Population Health, University of Oxford, Oxford, United Kingdom

**Keywords:** telomere, aging, whole exome sequencing, meta-analysis, ATM, RPL8

## Abstract

Telomeres are important for maintaining genomic stability. Telomere length has been associated with aging, disease, and mortality and is highly heritable (∼82%). In this study, we aimed to identify rare genetic variants associated with telomere length using whole-exome sequence data. We studied 1,303 participants of the Erasmus Rucphen Family (ERF) study, 1,259 of the Rotterdam Study (RS), and 674 of the British Heart Foundation Family Heart Study (BHF-FHS). We conducted two analyses, first we analyzed the family-based ERF study and used the RS and BHF-FHS for replication. Second, we combined the summary data of the three studies in a meta-analysis. Telomere length was measured by quantitative polymerase chain reaction in blood. We identified nine rare variants significantly associated with telomere length (*p*-value < 1.42 × 10^–7^, minor allele frequency of 0.2–0.5%) in the ERF study. Eight of these variants (in *C11orf65*, *ACAT1*, *NPAT*, *ATM*, *KDELC2*, and *EXPH5*) were located on chromosome 11q22.3 that contains *ATM*, a gene involved in telomere maintenance. Although we were unable to replicate the variants in the RS and BHF-FHS (*p*-value ≥ 0.21), segregation analysis showed that all variants segregate with shorter telomere length in a family. In the meta-analysis of all studies, a nominally significant association with LTL was observed with a rare variant in *RPL8* (*p*-*value* = 1.48 × 10^−6^), which has previously been associated with age. Additionally, a novel rare variant in the known *RTEL1* locus showed suggestive evidence for association (*p*-value = 1.18 × 10^–4^) with LTL. To conclude, we identified novel rare variants associated with telomere length. Larger samples size are needed to confirm these findings and to identify additional variants.

## Introduction

Telomeres are DNA structures located at the ends of chromosomes and consist of tandem hexanucleotide sequence repeats (TTAGGG) (Blackburn and Gall, 1978). They are important for maintaining genomic stability by preventing DNA degradation and chromosomal fusions (Blackburn, 2000). Telomeres are shortened with each cell division due to the inability of DNA polymerase to fully extend the 3′ end of the DNA strand during replication. When the telomeres reach a critical length, this leads to cellular senescence and ultimately to cell death, making them regulators of the replicative capacity of a cell and markers of biological age (Lindsey et al., 1991; Allsopp et al., 1992).

Shorter leukocyte telomere length (LTL) has been associated with several age-related diseases including cardiovascular diseases (Brouilette et al., 2003, 2007; Benetos et al., 2004; Fitzpatrick et al., 2007; Willeit et al., 2010a; Haycock et al., 2014), cancer (Hastie et al., 1990; Willeit et al., 2010b, 2011; Bojesen et al., 2013) and dementia (Martin-Ruiz et al., 2006; Grodstein et al., 2008; Honig et al., 2012). LTL has also been associated with mortality (Cawthon et al., 2003; Kimura et al., 2008; Fitzpatrick et al., 2011; Honig et al., 2012; Deelen et al., 2014; Rode et al., 2015; Marioni et al., 2016). However, this association has been inconsistent (Martin-Ruiz et al., 2005; Harris et al., 2006; Njajou et al., 2009; Houben et al., 2011; Strandberg et al., 2011; Needham et al., 2015). LTL is highly heritable with heritability estimates ranging from 34 to 82% (Slagboom et al., 1994; Bischoff et al., 2005; Vasa-Nicotera et al., 2005; Andrew et al., 2006; Broer et al., 2013). Previously, genome-wide association studies (GWASs) in European ancestry studies have identified common variants associated with LTL located in multiple genes, including: *TERC* (Codd et al., 2010, 2013; Mangino et al., 2012; Pooley et al., 2013), *TERT* (Codd et al., 2013; Pooley et al., 2013), *NAF1* (Codd et al., 2013), *OBFC1* (Levy et al., 2010; Mangino et al., 2012; Codd et al., 2013; Pooley et al., 2013), *RTEL1* (Codd et al., 2013), *CTC1* (Mangino et al., 2012), *ZNF676* (Mangino et al., 2012), *ZNF208* (Codd et al., 2013), *ACYP2* (Codd et al., 2013), *DCAF4* (Mangino et al., 2015), and *PXK* (Pooley et al., 2013). However, these variants explain < 5% of the heritability.

Up until now, no systemic whole exome or genome screen for rare variants has been published, despite the fact that these may explain part of the heritability (Manolio et al., 2009). Rare variants are not well captured by microarrays used for GWAS and remain difficult to impute, despite the recent improvements in imputation panels (McCarthy et al., 2016). Next generation sequencing technologies, such as whole-exome sequencing (WES), are better suited to study rare variants. In this study, we present a dual analysis. First, we conducted a genome-wide WES analysis of LTL in 1,303 individuals of the Dutch Erasmus Rucphen Family (ERF) study to search for rare genetic variants associated with LTL. The advantage of a family-based study is that the segregation of rare variants can be studied. We performed a replication analysis in the Rotterdam Study (RS) and the British Heart Foundation Family Heart Study (BHF-FHS). Next, we pooled the data together and conducted a meta-analysis of the association results of all three cohorts.

## Results

Descriptive statistics of the family-based and population-based studies are provided in Table 1. Mean age at LTL measurement was 49 years (SD = 15.0) in the ERF study and 61% of the study participants were female. The RS participants were older ($\bar{x}$_age_ = 75 years, SD = 7.7) and 57% of the participants were female, while mean age in the BHF-FHS was 58 years (SD = 8.2) and most study participants were male (26% female). Mean LTL values, measured in each participant using a quantitative polymerase chain reaction (qPCR) based technique as the ratio of telomere repeat length to copy number of the single copy gene *36B4*, were higher in the ERF study ($\bar{x}$_LTL_ = 1.85, SD = 0.35) than in the RS ($\bar{x}$_LTL_ = 0.94, SD = 0.18) and BHF-FHS ($\bar{x}$_LTL_ = 1.37, SD = 0.22). After adjusting LTL values for age and sex, mean LTL was comparable between studies (Table 1).

**TABLE 1:** Descriptive statistics of the study populations.

	ERF	RS	BHF-FHS
N	1303	1257	674
Mean age (SD)	48.9 (15.0)	74.5 (7.7)	58.0 (8.2)
Age range	18.2–95.7	55.0–105.8	36.0–81.0
% female	60.5	57.0	25.8
Mean LTL (SD)	1.85 (0.35)	0.94 (0.18)	1.37 (0.22)
LTL range	0.77–3.17	0.31–1.79	0.69–2.14
Adjusted mean	8.85 × 10^–18^ (0.32)	1.37 × 10^–17^ (0.18)	1.11 × 10^–10^ (0.21)
LTL (SD)*			
Adjusted LTL	−1.15–1.08	−0.71–0.89	−0.63–0.71
range*			

ERF = Erasmus Rucphen Family study; RS = Rotterdam Study; BHF-FHS = British Heart Foundation Family Heart Study; N = Number of variants; SD = Standard Deviation, LTL = Leukocyte Telomere Length. *LTL values were adjusted for age and sex and information on residuals is shown.

**FIGURE 1:**
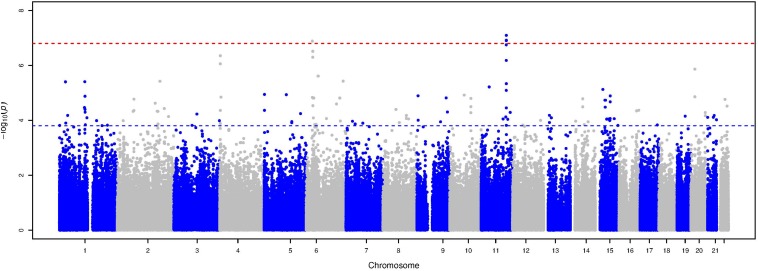
Manhattan plot of the association analysis with LTL in the ERF study. This plot shows –log10 transformed *p*-values (*y*-axis) for all variants present in the association analysis according to their position on each chromosome (*x*-axis). The red dashed line represents the Bonferroni corrected *p*-value threshold for significance (*p*-value < 1.42 × 10^–7^), while the blue dashed line represents the *p*-value threshold for suggestive significance (*p*-value ≤ 1.42 × 10^–4^).

**FIGURE 2:**
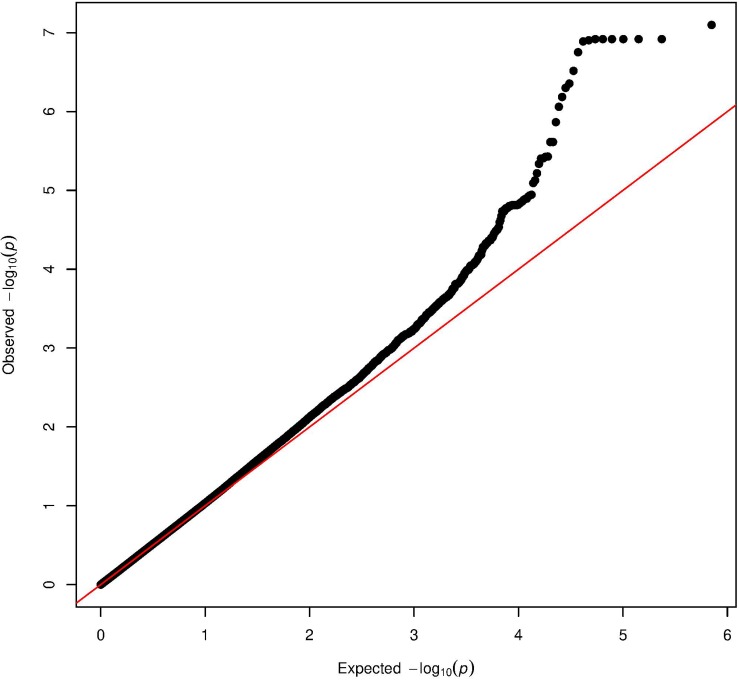
Quantile-quantile plot of the association analysis with LTL in the ERF study. The QQ-plot shows the observed test statistics (*y*-axis) plotted against the expected values of the test statistics (*x*-axis) (*X*^2^-distribution). The red line shows the distribution under the null hypothesis.

The Manhattan plot and the distribution of the test statistic [quantile-quantile (QQ) plot, λ = 1.04] of the WES analysis in the ERF study are presented in Figures 1, 2, respectively. We observed significant association of nine rare variants [Minor Allele Frequency (MAF) between 0.2% and 0.5%] with LTL as shown in Table 2. The significance threshold (*p*-value < 1.42 × 10^–7^) was adjusted for multiple testing using Bonferroni correction based on the number of variants in the analysis (0.05/353,075 variants). Each variant was negatively associated with LTL and the estimated effects of the minor allele of these variants were large (−2.18 < standardized β < −1.34), suggesting a significant decrease in LTL for each minor allele.

**TABLE 2:** Significant variants from the association analysis in the ERF study.

rsID	Gene	Chr	Position*	MAF	REF/ALT	GVS function**	PolyPhen score**	PhastCons score**	CADD score**	β	SE	*p*-value
rs185270276	*C11orf65*	11	108263828	0.005	T/C	Intron	Unknown	0	6.51	−1.34	0.25	7.99 × 10^–8^
rs12365364	*ACAT1*	11	108004687	0.005	G/A	Intron	Unknown	0	3.28	−1.38	0.26	1.21 × 10^–7^
rs79119325	*NPAT*	11	108032614	0.005	C/T	Missense	1	0.998	18.13	−1.38	0.26	1.21 × 10^–7^
rs3092910	*ATM*,*C11orf65*	11	108180917	0.005	T/C	Intron, synonymous	Unknown	0.997	9.87	−1.38	0.26	1.21 × 10^–7^
rs3218711	*ATM*,*C11orf65*	11	108236264	0.005	C/G	3-prime-UTR, intron	Unknown	0.002	5.56	−1.38	0.26	1.21 × 10^–7^
rs11212668	*KDELC2*	11	108352576	0.005	T/C	Intron	Unknown	0	1.44	−1.38	0.26	1.21 × 10^–7^
rs12146512	*EXPH5*	11	108384666	0.005	T/C	Missense	0.624	0.011	4.47	−1.38	0.26	1.21 × 10^–7^
rs2234993	*ATM*	11	108129599	0.005	C/G	Intron	Unknown	0	4.25	−1.37	0.26	1.25 × 10^–7^
rs144114619	*BTN3A1*	6	26408145	0.002	T/A	Missense	1	0.002	12.24	−2.18	0.41	1.29 × 10^–7^

Chr = Chromosome; MAF = Minor Allele Frequency; REF = Reference allele; ALT = Alternative allele; CADD = Combined Annotation Dependent Depletion; β = effect of the minor allele; SE = Standard Error. *Position according to Hg19. **Seattleseq Annotation Database 138.

**FIGURE 3:**
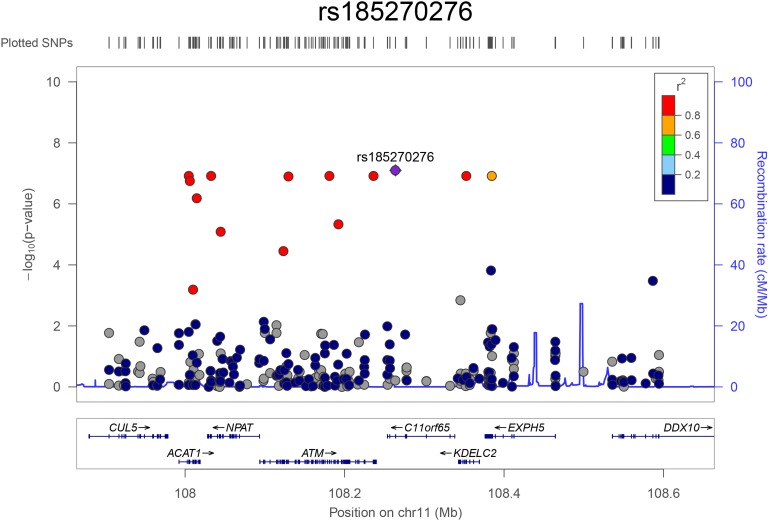
Regional association plot for the top hits on chromosome 11. The plot was constructed using LocusZoom (http://locuszoom.org/). The –log10 transformed *p*-values are plotted on the *y*-axis. The x-axis shows the position of the variants (dots) on chromosome 11 and the genes in this region. The most significant variant (rs185270276) is shown in purple and the color of the dots indicates the extend of linkage disequilibrium between the variant and the top variant.

**FIGURE 4:**
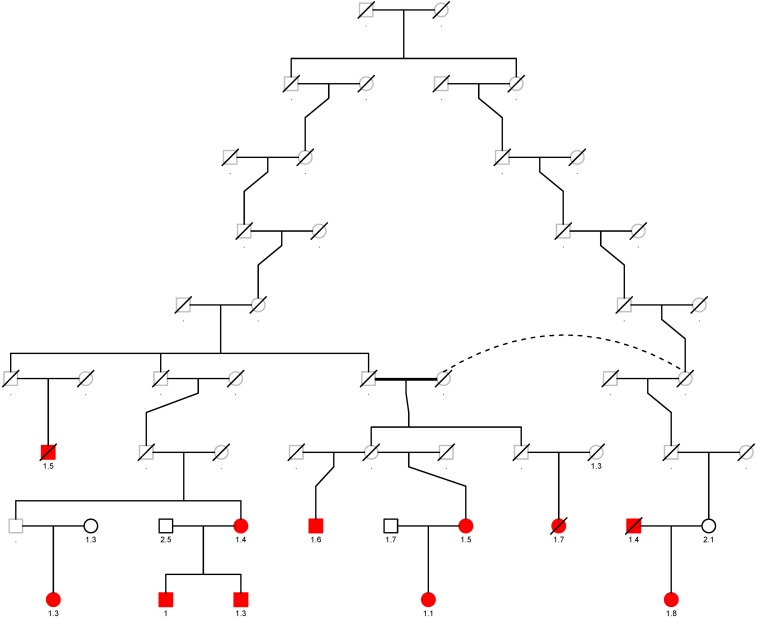
Segregation plot of the rare variants on chromosome 11q22.3 in the ERF study. The carriers of the rare variants located on chromosome 11q22.3 are depicted in red. The T/S ratio is added below the individuals of whom whole-exome sequencing data was available. Squares are males and rounds are females. Deceased individuals are denoted by a line through that specific individual. One person is twice in the pedigree, this is shown with the dotted line.

The top eight variants are located in a dense region on chromosome 11q22.3 (position range: 108004687 – 108384666, Figure 3) and appear to be a part of a haplotype that spans the *C11orf65*, *ACAT1*, *NPAT*, *ATM*, *KDELC2*, and *EXPH5* genes. A haplotype can describe a pair of genes inherited together from one parent on one chromosome, or it can describe all of the genes on a chromosome that were inherited together from a single parent. This haplotype segregates with shorter LTL in a family (Supplementary Figure 1), where it is carried by 14 individuals, 11 of whom were related within 4 generations according to the pedigree data (Figure 4). Further, the genetic kinship estimates show that the other three individuals are also related within 3–4 generations. These 8 variants are in strong linkage disequilibrium (pairwise LD: *r*^2^ between 0.93 and 1.00, D′ = 1) and show very similar *p*-values. The top variant rs185270276 is located in an intron of the *C11orf65* gene (MAF = 0.5%, β = −1.34, SE = 0.25, *p*-value = 7.99 × 10^–8^). The next six variants significantly associated with LTL (MAF = 0.5%, β = −1.38, SE = 0.26, *p*-value = 1.21 × 10^–7^) are located in the *ACAT1*, *NPAT*, *ATM*/*C11orf65*, *KDELC2*, and *EXPH5* genes. Two of these six variants are missense variants: rs79119325 (*NPAT*, PolyPhen = 1, CADD score = 18.1) and rs12146512 (*EXPH5*, PolyPhen = 0.624, CADD score = 4.5). The eighth significant variant, rs2234993, is located within an intron of *ATM* (MAF = 0.5%, β = −1.37, SE = 0.26, *p*-value = 1.25 × 10^–7^). The ninth significant variant, rs144114619 (MAF = 0.2%, *p*-value = 1.29 × 10^–7^), is a missense variant located on chromosome six in the *BTN3A1* gene, which is predicted to be damaging (PolyPhen = 1, CADD score = 12.2) and has the largest effect size (β = −2.18, SE = 0.41). There were six carriers of this variant in the ERF population. Interestingly, four of these carriers are also carriers of the rare variants in the chromosome 11q22.3 region (Supplementary Figure 2).

For replication analysis, we used WES data from two independent cohorts of European ancestry, the RS and the BHF-FHS. Results of the replication analysis are shown in Table 3, together with the results of the meta-analysis of summary statistics from all three cohorts for these variants. Six out of nine rare variants significantly associated with LTL in the ERF study (located in *ACAT1*, *NPAT*, *ATM/C11orf65*, *EXPH5*, and *BTN3A1*) were present in the RS and/or the BHF-FHS but were not significantly (*p*-value ≥ 0.21) associated with LTL (*p*-value < 0.025, 0.05/2 independent tests). The direction of effect of most variants was similar in the ERF study and the RS, while the BHF-FHS showed opposite direction of effect for most variants. Three variants, located in *C11orf65*, *KDELC2*, and *ATM*, were not present in the RS and BHF-FHS data. As these are unique to an isolated population, we were unable to confirm or reject their association with LTL in the replication cohorts.

**TABLE 3:** Replication results of the association analysis.

rsID	Gene	ERF (*N* = 1,303)				RS (*N* = 1,257)				BHF-FHS (*N* = 674)				Meta-analysis (*N* = 3,234)			
		MAF	β	SE	*p*-value	MAF	β	SE	*p*-value	MAF	β	SE	*p*-value	Direction*	β	SE	*p*-value
rs185270276	*C11orf65*	0.005	−1.34	0.25	7.99 × 10^–8^	–	–	–	–	–	–	–	–	–	–	–	–
rs12365364	*ACAT1*	0.005	−1.38	0.26	1.21 × 10^–7^	0.006	−0.20	0.25	0.43	0.010	0.30	0.26	0.25	−−+	−0.40	0.15	7.84 × 10^–3^
rs79119325	*NPAT*	0.005	−1.38	0.26	1.21 × 10^–7^	0.006	−0.24	0.28	0.39	0.010	0.30	0.26	0.25	−−+	−0.43	0.16	5.91 × 10^–3^
rs3092910	*ATM*,*C11orf65*	0.005	−1.38	0.26	1.21 × 10^–7^	0.007	−0.18	0.27	0.50	0.010	0.30	0.26	0.25	−−+	−0.41	0.16	8.67 × 10^–3^
rs3218711	*ATM*,*C11orf65*	0.005	−1.38	0.26	1.21 × 10^–7^	0.005	−0.24	0.28	0.39	0.010	0.30	0.26	0.25	−−+	−0.43	0.16	5.93 × 10^–3^
rs11212668	*KDELC2*	0.005	−1.38	0.26	1.21 × 10^–7^	–	–	–	–	–	–	–	–	–	–	–	–
rs12146512	*EXPH5*	0.005	−1.38	0.26	1.21 × 10^–7^	–	–	–	–	0.016	0.27	0.21	0.21	-?+	−0.38	0.17	2.61 × 10^–2^
rs2234993	*ATM*	0.005	−1.37	0.26	1.25 × 10^–7^	–	–	–	–	–	–	–	–	–	–	–	–
rs144114619	*BTN3A1*	0.002	−2.18	0.41	1.29 × 10^–7^	0.004	0.008	0.29	0.98	0.003	−0.43	0.48	0.38	-+-	−0.63	0.22	3.73 × 10^–3^

ERF = Erasmus Rucphen Family study; RS = Rotterdam Study; BHF-FHS = British Heart Foundation Family Heart Study; N = sample size; MAF = Minor Allele Frequency; β = Effect of the minor allele; SE = Standard Error. *Order of cohorts in direction column: ERF – RS – BHF-FHS; Direction of effect represented by − (negative association),+(positive association), or ? (not available).

Finally, to increase the statistical power, we performed an inverse-variance weighted meta-analysis of the association results from all three cohorts using METAL software. Variants were included in the meta-analysis if they were present in at least two out of three cohorts and had a minimum minor allele count of five in one or more cohorts, resulting in a multiple testing corrected significance threshold of 3.02 × 10^–7^ (0.05/165,311 variants). The top results of the meta-analysis (*p*-value < 3.02 × 10^–4^) are available in Table 4 and in Supplementary Table 1, which also contains cohort specific information. The Manhattan and QQ-plots are provided in Supplementary Figures 3, 4, respectively. The λ of 0.97 suggests the power has been low. Although there were no variants genome-wide significantly associated with LTL in the meta-analysis after adjusting for multiple testing, many of the top findings show a consistent effect across cohorts. The variant most significantly associated with LTL was a highly conserved synonymous variant (PhastCons score = 0.999) located in the *RPL8* gene on chromosome 8 (*p*-value = 1.48 × 10^–6^), which is predicted to be deleterious (CADD score = 15.11). Additionally, we used the meta-analysis results to perform a look-up of variants in loci identified by previous European ancestry GWASs (Supplementary Table 2). There were 153 variants present in these loci and we found suggestive evidence (*p*-value_meta_ < 3.02 × 10^–4^) for a positive association of a rare variant with LTL (rs181080831, β = 0.74, SE = 0.19, *p*-value = 1.18 × 10^–4^) in the known *RTEL1* locus.

**TABLE 4:** Suggestive findings of the meta-analysis (*p*-value ≤ 3.02 × 10^–4^).

												
rsID	Gene	Chr	Position*	REF/ALT	GVS function**	PolyPhen score**	PhastCons score**	CADD score**	β	SE	*p*-value	Direction^†^
8:146017422	*RPL8*	8	146017422	G/A	Synonymous	Unknown	0.999	15.11	1.93	0.40	1.48 × 10^–6^	+++
rs77919685	*LATS2*	13	21563311	G/A	Missense	0.002	0	7.001	0.44	0.09	2.23 × 10^–6^	+++
rs56041036	*ZFPM1*	16	88599023	A/G	Synonymous-near-splice	Unknown	0.998	10.77	–0.20	0.04	7.44 × 10^–6^	−−−
rs199779997	*MGA*	15	42058958	A/C	Missense	0.001	0.866	9.45	1.77	0.40	8.60 × 10^–6^	+++
rs4895944	*VNN2*	6	133070995	G/T	Missense, non-coding-exon	0.038	0.003	16.69	–1.29	0.29	8.99 × 10^–6^	−−?
rs7735563	*RAPGEF6*	5	130764936	T/C	Intron	Unknown	1	19.08	–0.77	0.18	1.53 × 10^–5^	−−−
rs189691392	*SF3B5*	6	144416667	G/A	5-prime-UTR	Unknown	0	7.119	–1.82	0.42	1.77 × 10^–5^	-?-
rs138765444	*ZKSCAN4*	6	28219377	A/G	Synonymous, intron	Unknown	0.036	9.324	–1.27	0.30	2.59 × 10^–5^	−−−
rs1783091	*none*	21	33964605	T/C	Intergenic	Unknown	0.004	0.154	0.22	0.05	2.67 × 10^–5^	+?+
rs2170177	*DOCK3*	3	51349887	C/A	Intron	Unknown	0	0.044	–0.36	0.09	5.06 × 10^–5^	−−−
rs5930	*LDLR*	19	11224265	A/G	Synonymous	Unknown	0.001	0.579	–0.10	0.03	5.73 × 10^–5^	−−−
rs55648406	*TUB*	11	8060566	G/A	Missense, intron	0.086	1	15.76	–0.62	0.16	5.85 × 10^–5^	−−−
rs140456008	*SLC35G2*	3	136574420	A/G	Missense	0.941	1	10.84	1.89	0.47	6.67 × 10^–5^	++?
rs139380413	*COL8A1*	3	99513830	G/A	Missense	0.071	0.966	11.23	–1.26	0.32	7.88 × 10^–5^	−−−
rs11656725	*LRRC48*	17	17900726	C/T	Intron	Unknown	0	0.471	0.38	0.10	8.25 × 10^–5^	+?+
rs7193541	*RFWD3*	16	74664743	T/C	Missense	0.008	0.485	9.1	–0.10	0.03	8.28 × 10^–5^	−−−
rs17222435	*SLC28A1*	15	85488335	C/T	Intron	Unknown	0	3.598	0.69	0.18	9.07 × 10^–5^	+++
rs117223521	*none*	8	38964715	T/C	Upstream-gene	Unknown	0	1.194	–1.36	0.35	1.01 × 10^–4^	-?-
rs11700220	*MTG2*	20	60770931	A/G	Missense	1	1	21.6	0.47	0.12	1.03 × 10^–4^	+++
rs187466877	*GPN1*	2	27862872	A/G	Intron	Unknown	0	1.47	0.85	0.22	1.05 × 10^–4^	+?+
rs56188826	*MARK1*	1	220791870	C/T	Synonymous	Unknown	0.123	6.414	0.41	0.11	1.14 × 10^–4^	+?+
rs1872592	*PIF1*	15	65113493	G/A	Intron	Unknown	0	0.005	–0.10	0.02	1.15 × 10^–4^	−−−
rs73056605	*CLEC4C*	12	7894056	G/A	Missense	0.037	0	0.005	0.11	0.03	1.18 × 10^–4^	+++
rs181080831	*RTEL1*, *RTEL1-TNFRSF6B*	20	62326874	G/A	Intron, non-coding-exon, synonymous	Unknown	0	4.04	0.74	0.19	1.18 × 10^–4^	+++
rs13014800	*CENPA*	2	27015118	G/A	Intron	Unknown	0.163	11.67	–0.13	0.03	1.23 × 10^–4^	-?-
rs143463783	*TRIM27*	6	28889741	G/A	Synonymous	Unknown	1	9.216	–1.22	0.32	1.24 × 10^–4^	-?-
rs374215951	*PIEZO1*	16	88788318	G/A	Synonymous	Unknown	0.21	0.893	–2.57	0.67	1.31 × 10^–4^	-?-
rs181215404	*EPPK1*	8	144941659	G/A	Synonymous	Unknown	0.011	4.835	0.94	0.25	1.32 × 10^–4^	+?+
rs377359525	*FTCD*	21	47572869	A/G	Missense	1	1	16.44	1.68	0.44	1.33 × 10^–4^	+++
rs10936599	*MYNN*	3	169492101	C/T	Synonymous, non-coding-exon, 5-prime-UTR	Unknown	1	10.1	–0.11	0.03	1.38 × 10^–4^	−−−
rs74730846	*STXBP5L*	3	120924764	C/T	Intron-near-splice	Unknown	0.629	5.818	–0.19	0.05	1.39 × 10^–4^	-?-
rs137853096	*HSD17B4*	5	118788316	G/A	Missense, 5-prime-UTR	1	1	24	–0.73	0.19	1.67 × 10^–4^	−−?
rs41284136	*IFIT3*	10	91087805	G/C	5-prime-UTR	Unknown	0	7.796	0.40	0.11	1.76 × 10^–4^	+++
rs58106741	*SLC4A1AP*	2	27886820	G/T	Synonymous	Unknown	0	5.939	0.70	0.19	1.80 × 10^–4^	+?+
rs58068845	*UTP6*	17	30200363	G/A	Intron	Unknown	0	4.223	0.35	0.09	1.90 × 10^–4^	+++
rs200602887	*GREB1*	2	11751072	G/C	Synonymous	Unknown	0.986	10.5	–1.02	0.27	2.01 × 10^–4^	−−−
rs141180155	*LRP2*	2	170127559	G/A	Synonymous, intron	Unknown	0	13.06	–0.36	0.10	2.08 × 10^–4^	−−−
rs7837242	*LONRF1*	8	12600622	C/T	Intron	Unknown	0.001	4.941	–0.18	0.05	2.08 × 10^–4^	-?-
rs115018606	*C2orf16*	2	27799773	A/C	Missense	0.972	0.002	5.869	0.41	0.11	2.12 × 10^–4^	+?+
rs151309008	*REXO2*	11	114310345	C/T	Missense	0.437	1	17.76	–0.64	0.17	2.12 × 10^–4^	−−−
rs143759519	*PYGL*	14	51382637	G/A	Missense	1	0.975	34	0.55	0.15	2.13 × 10^–4^	+++
rs10936600	*LRRC34*	3	169514585	A/T	Missense	1	0.001	12.07	–0.11	0.03	2.14 × 10^–4^	−−−
rs117178504	*DYNC2H1*	11	103153788	C/A	Synonymous	Unknown	0.996	8.615	0.38	0.10	2.18 × 10^–4^	+++
rs367644268	*COA5*	2	99224742	C/T	Intron	Unknown	0.015	8.626	–1.42	0.39	2.28 × 10^–4^	−−?
rs146979490	*GPN1*	2	27864089	A/G	Intron	Unknown	0.025	11.52	0.51	0.14	2.43 × 10^–4^	+++
rs146033252	*MTA3*, *OXER1*	2	42991127	G/A	Missense, intron	0.084	0.023	8.404	0.71	0.19	2.48 × 10^–4^	+++
rs141280036	*PADI4*	1	17634718	A/G	Missense	0.992	0.881	13.72	0.79	0.22	2.51 × 10^–4^	+++
rs79400176	*DZANK1*	20	18414309	C/T	Missense	0.129	0.994	2.801	–0.36	0.10	2.55 × 10^–4^	−−−
rs116604207	*RBM5*	3	50147061	G/A	Synonymous, non-coding-exon	Unknown	0.453	11.12	0.79	0.22	2.62 × 10^–4^	+++
rs41307740	*LBR*	1	225601614	C/A	Intron	Unknown	0.004	5.956	–0.65	0.18	2.76 × 10^–4^	-?-
rs369623673	*ELAC2*	17	12899160	C/T	Intron	Unknown	0	6	0.93	0.26	2.77 × 10^–4^	+?+
rs9997727	*C4orf50*	4	5969113	G/A	Intron	Unknown	0	0.613	–0.10	0.03	2.85 × 10^–4^	−−−
rs150538926	*PDZD2*	5	32037369	C/T	Synonymous	Unknown	0	0.482	0.40	0.11	2.87 × 10^–4^	+++
rs55868421	*TRIM5*	11	5688948	A/G	Intron-near-splice, intron	Unknown	0	5.512	0.44	0.12	2.94 × 10^–4^	+++
rs323895	*ACY1*, *ABHD14A- ACY1*	3	52021316	A/G	Intron	Unknown	0	1.818	–0.36	0.10	3.00 × 10^–4^	−−−
rs7188880	*RFWD3*	16	74664810	A/T	Synonymous	Unknown	1	10.78	–0.09	0.02	3.01 × 10^–4^	−−−

Chr = Chromosome; REF = Reference allele; ALT = Alternative allele; CADD = Combined Annotation Dependent Depletion; β = effect of the minor allele; SE = Standard Error. *Position according to Hg19. **Seattleseq Annotation Database 138. ^†^Order of cohorts in direction column: ERF, RS, BHF-FHS; Direction of effect represented by − (negative association) + (positive association) or ? (not available).

## Discussion

In the family-based ERF study, we identified nine rare variants (MAF between 0.2% and 0.5%) associated with LTL by performing a WES association analysis. The eight most significantly associated variants are located in a region on chromosome 11q22.3 and segregate together with shorter LTL in one family. This region contains the *ATM* locus that has previously been shown to be involved in telomere maintenance and genomic stability and is thus an obvious candidate gene. In the meta-analysis of discovery and replication cohorts, we identified another rare missense variant in the *RPL8* gene strongly associated with LTL. Although we were not able to replicate either of the associations, both *ATM* and *RPL8* have been previously found to be strong predictors of telomere length (*ATM*) and chronological age (*ATM* and *RPL8*).

Interestingly, we identified three unique rare variants in the *ATM* (Ataxia Telangiectasia Mutated) gene associated with LTL in the ERF study. *ATM* is the homolog of the *Tel1* gene in yeast (Greenwell et al., 1995) and has been implicated in important telomere maintenance processes (Pandita, 2002; Lee et al., 2015; Tong et al., 2015). The ATM protein kinase is a master controller of cell cycle checkpoint signaling pathways required for cell response to DNA damage and for genomic stability (https://www.ncbi.nlm.nih.gov/gene/?term=472). Additionally, ATM kinase is necessary for telomere elongation (Lee et al., 2015; Tong et al., 2015). *ATM* is involved in the genetic disorder ataxia telangiectasia (AT), which is characterized by cerebellar ataxia, oculocutaneous telangiectasia, immunodeficiency, and a predisposition to cancer (Savitsky et al., 1995). Cells of AT patients also show telomeric fusions and have accelerated telomere shortening with increasing age (Metcalfe et al., 1996). Furthermore, *ATM* was significantly associated with chronological age in a meta-analysis of gene expression profiles, showing lower transcript abundance in older individuals (Peters et al., 2015). Although genetic variants in *ATM* have been associated with various cancers (Barrett et al., 2011; Helgason et al., 2015; Ransohoff et al., 2017; Scelo et al., 2017), only one genetic variant in *ATM*, rs227080, was genome-wide significantly associated with LTL in a Singaporean Chinese population (Dorajoo et al., 2019). This variant was not significantly associated with LTL in the ERF study, the Rotterdam Study or the BHF-FHS, implicating it was not driving the association observed in the current study.

Segregation analysis of the variants within the chromosome 11q22.3 region in the ERF study showed that the variants segregate with shorter LTL in one family. We did not detect a specific disease that segregates in this family, which may be explained by the relatively young age of most of the carriers. In the replication analysis, we were not able to confirm the association of the nine variants associated with LTL in the RS and BHF-FHS, due to lack of association (six variants) or absence of the variant in the replication cohorts (three variants). However, it is possible this signal comes from variants that are family-specific and thus may not be transferable to the general population. Likewise, it may also be that the whole haplotype has an effect on telomere length, an haplotype that is most likely unique to the isolated ERF population. At this locus, the *ATM* gene is currently the most likely causative gene. Lastly, we performed a meta-analysis of the three cohorts. The top variant of the meta-analysis is located in *RPL8* (Ribosomal Protein L8). This is interesting as *RPL8*, together with six other *RPL* genes, was negatively associated with chronological age (Peters et al., 2015). In this transcriptomic study, Peters et al. identified 1,497 genes whose expression level changes associated with age; this gene list includes *RPL8* and *ATM*. We additionally found suggestive evidence for an association of a novel rare variant in the known *RTEL1* locus. It would be interesting to perform a WES or whole-genome sequencing meta-analysis with larger sample size to increase the statistical power.

The advantage of our study is that we used a family-based setting that allowed us to show segregation of the variants located on chromosome 11q22.3 in a family. The ERF study population has shown a low immigration rate and a high level of inbreeding, which has increased the frequency of many rare alleles (Pardo et al., 2005). Another advantage is that all three studies used the standardized qPCR method to quantify LTL. However, there are also several limitations to our study. The first limitation is that our findings in the ERF study are not easy to generalize to the general population as these findings may be family-specific. However, we identified genes that are known to be related to telomere processes and thus are plausible candidate genes. The second limitation of our study may be that we used blood measurements of telomere length instead of tissue specific measurements, although previous studies have shown that mean telomere length in blood and other tissues are highly correlated (Okuda et al., 2002; Wilson et al., 2008) and, therefore, we expect this did not influence our findings. The third limitation is the difference in LTL distribution between the three studies, which may be explained by the different age distributions in the studies. Mean age is lowest in the ERF study (49 years), highest in the RS (75 years) and mean age in the BHF-FHS is in between (58 years). As LTL decreases significantly with age (Lindsey et al., 1991; Slagboom et al., 1994; Blackburn, 2001) and is associated with mortality (Cawthon et al., 2003; Kimura et al., 2008; Fitzpatrick et al., 2011; Honig et al., 2012; Deelen et al., 2014; Rode et al., 2015; Marioni et al., 2016), the variation in LTL becomes less with the aging populations. Mean LTL values were comparable between studies after adjusting LTL values for age and sex. To further standardize across the three cohorts, a z-transformation of the LTL values was performed resulting in comparable distributions with mean of zero and standard deviation of one. Nevertheless, these differences between studies, together with the small sample size of the BHF-FHS cohort, could potentially explain the lack of replication. The fourth limitation is that we were unable to calculate the effect of the variants on telomere length in base pairs because of the quantification method of LTL in our study together with the z-transformation that was applied to the LTL values to standardize across the cohorts.

To conclude, this first study using WES data to search for rare genetic variants associated with LTL has identified interesting variants and genes associated with shorter LTL. Eight out of nine rare variants associated with LTL are located on chromosome 11q22.3 and all variants segregate within an ERF family. As we were not able to replicate findings, future studies should further investigate this region and the other genes identified in this study to confirm their involvement in telomere length regulation.

## Materials and Methods

### Study Populations

Our discovery population consisted of participants from the family-based Erasmus Rucphen Family (ERF) study. The ERF study comprises approximately 3,000 inhabitants of a recent genetically isolated community in the Southwest of the Netherlands, studied as part of the Genetic Research in Isolated Population program (Pardo et al., 2005). All ERF participants are descendants of 22 founder couples who had at least six children baptized in the community church in the 18th century, or their spouses. Baseline data collection, including blood withdrawal, took place between 2002 and 2005. As the ERF population shows a low rate of immigration and a high level of inbreeding, the frequency of several rare alleles is increased in this population (Pardo et al., 2005). The ERF study was approved by the Medical Ethics Commitee of the Erasmus Medical Center (MC), Rotterdam, Netherlands. All participants provided written informed consents and all investigations were carried out in accordance with the Declaration of Helsinki.

The replication cohorts included participants from the Rotterdam Study (RS) and the British Heart Foundation Family Heart Study (BHF-FHS). The RS is a prospective cohort study ongoing since 1990 in the well-defined Ommoord district in Rotterdam, The Netherlands. The original RS cohort (RS-I) included 7,983 individuals of 55 years of age or over. At baseline, participants were interviewed at home and had an extensive set of examinations, which were repeated every 3–4 years (Ikram et al., 2017). The Rotterdam Study has been approved by the Medical Ethics Committee of the Erasmus MC and by the Dutch Ministry of Health, Welfare and Sport. The Rotterdam Study has been entered into the Netherlands National Trial Register (NTR; www.trialregister.nl) and into the WHO International Clinical Trials Registry Platform (ICTRP; www.who.int/ictrp/network/primary/en/) under shared catalogue number NTR6831. All participants provided written informed consent to participate in the study and to have their information obtained from treating physicians.

The British Heart Foundation Family Heart study recruited families with at least two siblings diagnosed with premature (<66 years) coronary artery disease (CAD) within the United Kingdom between 1998 and 2003. Full details are provided elsewhere (Samani et al., 2005, 2007).

### Telomere Length

Mean LTL values in the ERF study and the BHF-FHS study were measured using a qPCR method in all samples (Cawthon, 2002). The measurements were performed in Leicester, United Kingdom, and details of the measurements were previously described (Codd et al., 2010, 2013). In summary, mean LTL was measured in leukocytes and expressed as the ratio (T/S ratio) of telomere repeat length (T) to the copy number of a single-copy gene, *36B4* (S). Samples were quantified relative to a calibrator sample used on each run (DNA from the K562 cell line) (Codd et al., 2010).

In the RS, mean LTL values were also measured using a qPCR assay based on the method described by Cawthon (2002) with minor modifications. For each sample the telomere and *36B4* assay were run in separate wells but in the same 384 wells PCR plate. Each reaction contained 5 ng DNA, 1 uM of each of the telomere primers (tel1b-forward: GGTT TGTTTGGGTTTGGGTTTGGGTTTGGGTTTGGGTT, tel2b- reverse: GGCTTGCCTTACCCTTACCCTTACCCTTACCCTTA CCCT) or 250 nM of the *36B4* primers (36B4u-forward: CAG CAAGTGGGAAGGTGTAATCC, 36B4d-reverse: CCCATTCT ATCATCAACGGGTACAA) and 1x Quantifast SYBR green PCR Mastermix (Qiagen). The reactions for both assays were performed in duplicate for each sample in a 7900HT machine (Applied Biosystems). Ct values and PCR efficiencies were calculated per plate using the MINER algorithm (Zhao and Fernald, 2005). Duplicate Ct values that had a Coefficient of Variance (CV) of more than 1% were excluded from further analysis. Using the average Ct value per sample and the average PCR efficiency per plate the samples were quantified using the formula Q = 1/(1 + PCR eff)^Ct. The relative telomere length was calculated by dividing the Q of the telomere assay by the Q of the *36B4* assay. To validate the assay 96 random samples were run twice and the CV of that experiment was 4.5%.

### Exome Sequencing

The exomes of 1,336 ERF participants were sequenced at the Erasmus Center for Biomics of the department of Cell Biology, Erasmus MC, The Netherlands. The exomes of a randomly selected subset of 2,628 individuals from the RS-I population were sequenced at the Human Genotyping facility of the Department of Internal Medicine, Erasmus MC, The Netherlands. Details of the methods and quality control for ERF and RS are described elsewhere (Amin et al., 2016; van Rooij et al., 2017). In total, 1,303 ERF participants and 1,257 RS participants had both exome sequence and LTL data available and were included in this analysis. A subset of the BHF-FHS, comprising of 674 unrelated individuals of Caucasian ancestry who had previously undergone exome sequencing as part of the Leicester Myocardial Infarction Study (Khera et al., 2017) and had LTL data available (Codd et al., 2010) were included in this analysis.

### Statistical Analyses

For each individual cohort quantitative trait association analysis was performed using Rare Variant tests (RVtests) software, which supports the analysis of related individuals (Zhan et al., 2016). Association analysis was performed using a score test, assuming an additive model, suitable for analysis with related and unrelated individuals. We applied a z-transformation of LTL values for the three cohorts separately to standardize values across cohorts. All analyses were adjusted for age, sex and batch effects (if needed). Furthermore, we adjusted for familial relationships in ERF using the kinship matrix estimated from the genotyped data, while in the RS we corrected for the first four principal components as the fourth principal component was significantly associated with LTL. Only variants with a minor allele count ≥ 5 were included.

In the ERF study, we calculated the pairwise LD (*r*^2^ and D′) between the top eight variants on chromosome 11 that were significantly associated with LTL using the –ld command of PLINK 1.9 software (Chang et al., 2015) (www.cog-genomics.org/plink/1.9/). Additionally, we performed an inverse-variance weighted meta-analysis using METAL software (Willer et al., 2010). In the analysis using data of the family-based ERF study, we corrected the significance thresholds for multiple testing using Bonferroni correction, resulting in a significance threshold of 1.42 × 10^–7^ (0.05/353,075). In the replication analysis, the multiple testing corrected *p*-value thresholds was 0.025 (0.05/2 independent tests). In the meta-analysis, we adjusted for the number of variants tested, resulting in a significance threshold of 3.02 × 10^–7^ (0.05/165,311).

## Data Availability Statement

The datasets analyzed for each individual cohort can be requested by contacting the responsible Principal Investigator. Because of restrictions based on privacy regulations and informed consent of the participants, data cannot be made freely available in a public repository. For the Rotterdam Study data, requests should be directed toward the management team of the Rotterdam Study (secretariat.epi@erasmusmc.nl), which has a protocol for approving data requests.

## Ethics Statement

The studies involving human participants were reviewed and approved by their respective Medical Ethics Boards and all investigations were carried out in accordance with the Declaration of Helsinki. The patients/participants provided their written informed consent to participate in this study.

## Author Contributions

AS, NA, and CD designed the study and wrote the manuscript. AS, SW, CN, and SA performed the analyses. LB, PA, RB, MD, MH, JR, RK, and NA were involved in the data collection and provided the data. WI, NS, MI, AU, and CD were involved in the supervision of individual cohorts. AS, SW, CN, DV, NS, VC, NA, and CD interpreted the results. All authors critically reviewed and approved the manuscript.

## Conflict of Interest

AS is an employee of the company SkylineDx. The remaining authors declare that the research was conducted in the absence of any commercial or financial relationships that could be construed as a potential conflict of interest.
