# Supplementary material for: Exome Sequencing Analysis Identifies Rare Variants in ATM and RPL8 That Are Associated With Shorter Telomere Length
Source: Front Genet. 2020 Apr 30;11:337. doi: 10.3389/fgene.2020.00337 (PMC7204400; doi:10.3389/fgene.2020.00337)
Supplement: Supplementary file 2 [file Data_Sheet_2.docx]

# **Supplementary Figures**


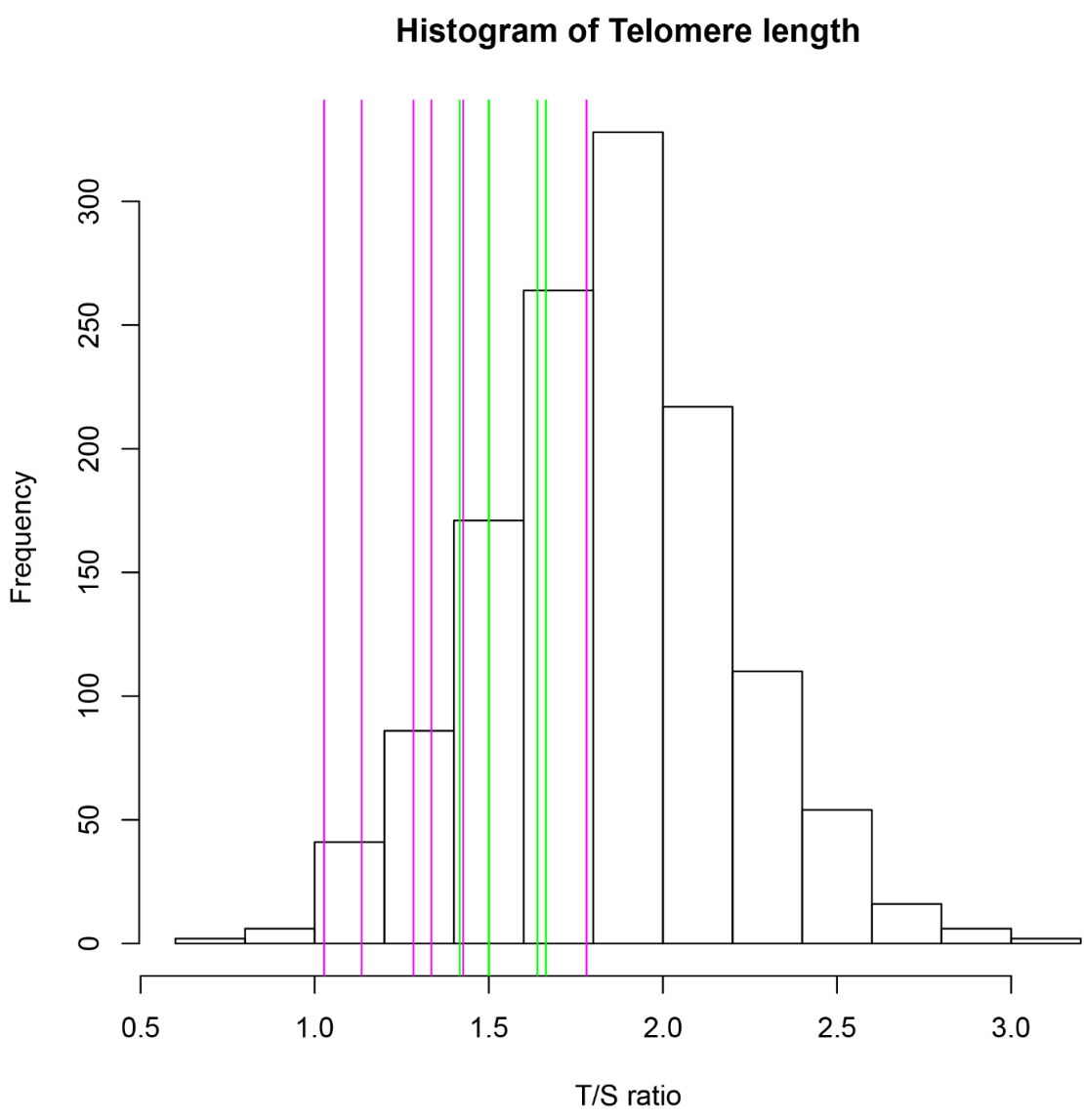


**Supplementary Figure 1.** Histogram of LTL in 1,303 participants of the ERF study marking the carriers of the haplotype on chromosome 11q22.3.

The magenta lines indicate the carriers that are younger than 48.9 years (mean age of the ERF study participants in the analysis) and the green lines indicate the carriers that are older than 48.9 years.


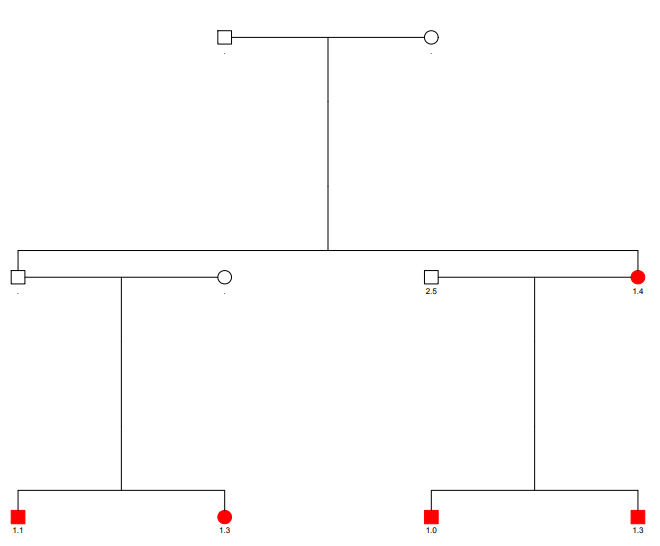


**Supplementary Figure 2.** Segregation plot of the rs144114619 variant (*BTN3A1*) in the ERF study.

The carriers of the rs144114619 variant located in the BTN3A1 gene on chromosome 6 are depicted in red. The T/S ratio is added below the individuals of whom whole-exome sequencing data was available.


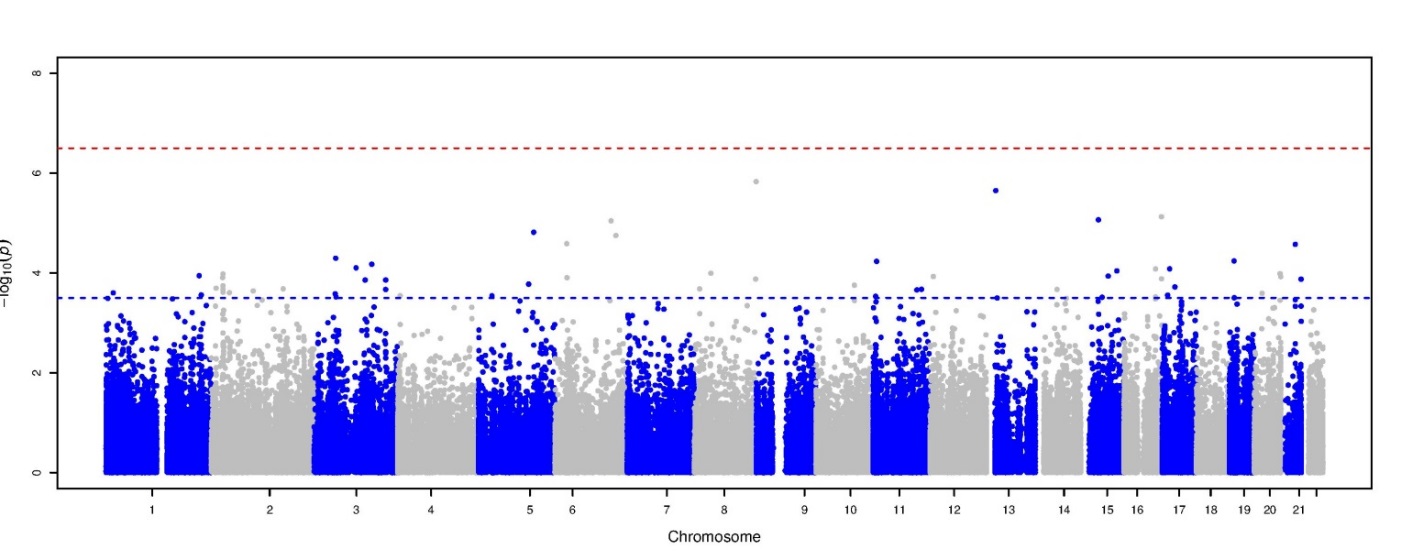


**Supplementary Figure 3.** Manhattan plot of the association analysis with LTL in the meta-analysis.

This plot shows –log10 transformed *p*-values (y-axis) for all variants present in the single variant meta-analysis according to their position on each chromosome (x-axis). The red dashed line represents the Bonferroni corrected *p*-value threshold for significance (*p*-value < 3.02x10^-7^), while the blue dashed line represents the *p*-value threshold for suggestive significance (*p*-value < 3.02x10^-4^).


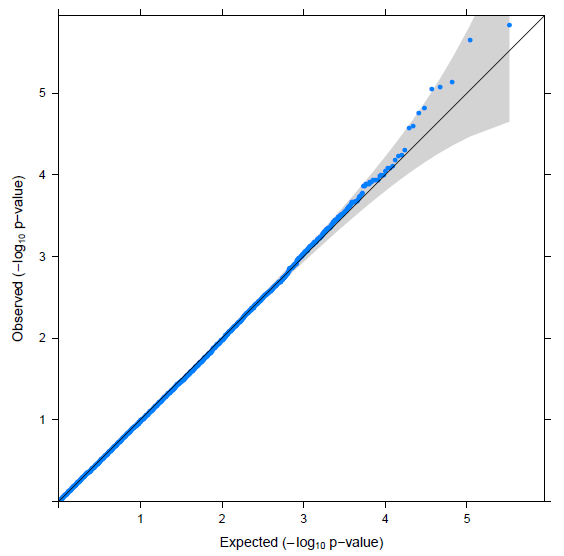


**Supplementary Figure 4.** Quantile-quantile plot of the association analysis with LTL in the meta-analysis.

The QQ-plot shows the observed test statistics (y-axis) plotted against the expected values of the test statistics (x-axis) (Χ^2^-distribution). The black line shows the distribution under the null hypothesis. The grey area represents the 95% confidence interval.
